# Ocular Torsion in Children with Horizontal Strabismus or Orthophoria †

**DOI:** 10.3390/children10091536

**Published:** 2023-09-11

**Authors:** Nayrouz Bdeer, Noa Hadar, Doris Raveh, Basel Obied, Stephen Richard, Alon Zahavi, Nitza Goldenberg-Cohen

**Affiliations:** 1 Faculty of Medicine, Hadassah Hebrew University, Jerusalem 91120, Israel; nayrouz.bdeer@gmail.com; 2 Bnai-Zion Medical Center, Ophthalmology Department, Haifa 3339419, Israel; noa.hadar@b-zion.org.il (N.H.); doris.raveh@b-zion.org.il (D.R.); 3 The Krieger Eye Research Laboratory, Bruce and Ruth Faculty of Medicine, Technion Institute of Technology, Haifa 3200003, Israel; basel.obied01@gmail.com (B.O.); steverit@campus.technion.ac.il (S.R.); 4 Ophthalmology Department and Laboratory of Eye Research, Felsenstein Medical Research Center, Rabin Medical Center, Petach Tikva 4917002, Israel; alonzahavi@gmail.com; 5 Sackler Faculty of Medicine, Tel Aviv University, Tel Aviv 69978, Israel

**Keywords:** strabismus, torsion, fundus photos, strabismus surgery

## Abstract

Purpose: To report the rate of ocular torsion in children with horizontal strabismus or orthophoria. Methods: A retrospective study design was used. Nineteen children were included in the study, including seven girls, aged 4–16 years. All patients were examined for strabismus and 12 were scheduled for surgical intervention. All participants had digital fundus photos (DRSplus, Padova, Italy) of both eyes at presentation, and 5 of 12 also had fundus photos following the strabismus operation. Patient files were reviewed for age, demographic data, type of strabismus, clinical symptoms and signs, orthoptic exams, subjective and objective reports of torsion, inferior oblique overaction, and V pattern. Fundus photos were analyzed for torsion by ImageJ software [ImageJ 1.54f, National Institute of Health, USA]. The disc-foveal angle was calculated for ocular torsion. Disc-foveal angle was defined as the angle formed between a line passing through the center of the optic disc to the fovea and another horizontal line passing through the center of the optic disc, using fundus photographs. Results: Of the 19 children, 18 had horizontal strabismus: 9 with exotropia and 9 with esotropia. One child was orthophoric with torsional strabismus. Inferior oblique overaction was detected in all but 3 children, while V pattern was documented in 10. Visual acuity was reduced (under 6/12) in four eyes of four children. None were symptomatic for ocular torsion. Although extorsion was documented clinically in 3 of 19 children, it was measurable on fundus photos in all patients before surgery with a mean of 8.7 ± 8.5 degrees and 8.5 ± 9.7 degrees in the right and left eyes, respectively. The mean extorsion in both eyes was 19.7 ± 10.1 degrees and improved to a mean of 15.3 ± 7.9 degrees in the children who were operated on and had documented postoperative fundus photographs. Conclusions: Extorsion was detected on fundus photos at a significantly higher rate than in clinical examination. Notably, inferior oblique overaction was mainly associated with torsion. This study demonstrated that torsion is underdiagnosed in clinical examinations, as the children are often asymptomatic, but fundus photos which are easily obtained can improve its detection.

## 1. Introduction

Horizontal strabismus is common among children. The most common type is congenital or infantile esotropia with absence of binocular vision which is sometimes accompanied by amblyopia. The incidence of acquired exotropia increases during childhood. Both types of horizontal strabismus, exodeviation or esodeviation, may present with an A or V pattern, secondary to the unbalanced activity of the oblique muscles. Torsional strabismus refers to a twisting rotation around the visual axis [1].

V pattern strabismus Is defined when the horizontal measurements of the deviation differ between upgaze to downgaze by at least 15 prism diopters (PD). It is reported to accompany horizontal strabismus in up to 50% of cases [2]. The most common underlying etiology of V pattern strabismus is overaction of the inferior oblique muscles. This can lead to additional torsional effects on the already horizontally drifted eye. Therefore, strabismus surgery involving a horizontal muscle can be supplemented by additional surgery of the inferior obliques when overaction leads to a V/A pattern.

Clinical assessment of torsion, subjective or objective anatomic torsion, is difficult in all ages, especially in children. Subjective methods are numerous and include double Maddox test, synoptophore, Gracis torsionometer, Harms screen, and Awaya cyclodeviation test [3]. Clinical assessment might also be influenced by the head position. However, objective measurements are vital in order to reach an accurate diagnosis and make a surgical decision regarding the treatment of the cyclovertical strabismus [4]. Commonly suggested methods for objective assessment of torsion include measurement of the ocular torsion angle [5]. It is recommended to use the optic disc–macula relationship, by imagining a line relative to the horizontal line, during fundus exam by indirect ophthalmoscopy. The average location of the fovea in relation to the optic nerve was found to be 0.3 disc diameters below a horizontal line extended through the geometric center of the optic disc [4]. A shadow from a target on the macula can assist in identifying the center of vision [5]. This clinical method is difficult to document, and therefore possesses inherent limitations [5]. Use of retinal vascular clues was suggested to improve the exam [5]. Identification of the axis of the retinal vascular arcades was found to aid as a direct and accessory means to provide additional pertinent information in diagnosing ocular cyclorotations [5]. Fundus photography can overcome most of the difficulties, is taken within seconds and is considered a reliable method to document and evaluate the torsion objectively [6].

The cycloversion mechanism controlled by vision is used to maintain clear vision and binocular single vision [7]. This mechanism is mainly controlled by the vestibular system and relates to retinal image stabilization during head movements [7]. Although human binocular vision can demonstrate the sensory capacity for cyclofusion beyond 10°, torsional disparity of 6° or more significantly degrades horizontal fusional vergence and stereopsis [8]. Under pathological conditions, asymptomatic torsion is reported with a prevalence of 94% in patients with acute lesions of the brainstem [9], compared to symptomatic torsion of 60% in patients with acquired superior oblique palsy. In healthy adults, as the subjective cyclofusion range decreases with age, the incidence of extorsion seems to increase [10]. However, adults and children rarely complain of torsion. In clinical exams, torsion is often underdiagnosed. Recently, with the use of digital photos, torsional changes before and after lateral rectus recession for intermittent exotropia in children were reported [11]. The significant change in ocular torsion suggested that ocular torsion might be influenced by fusional control and may predict the response to exotropia strabismus surgery [11].

Torsion was first described by von Graefe who noted a vertical macular displacement with respect to the optic disc [12]. However, ocular torsion is often not considered in the clinical diagnosis of ocular motility disorders nor taken into the preoperative surgical measurement calculations for horizontal strabismus surgery [1]. As fundus exam of children can be difficult, measuring a torsional component may be neglected. The common practice relies on subjective torsional measurements, but currently, fundus photos can be used to improve objective torsion measurements in each eye. Although not often used in clinical practice, such photos are relatively easy to acquire, even in children.

The aim of this study was to compare subjective and objective torsion symptoms and signs in pediatric horizontal strabismus using digital fundus photos. In children who have undergone strabismus surgery, we also aimed to compare the effect of surgery on torsion measurements. The results of the study may benefit future pediatric preoperative clinical strabismus examinations, influence surgical planning, and improve clinical outcomes.

## 2. Methods

A retrospective cohort study design was used. Patients’ files were reviewed for demographic, orthoptics, and clinical ophthalmology exams at presentation and the end of follow-up. Fundus photos taken by DRSplus fundus camera (Padova, Italy) were documented in the medical files of all patients at least once at presentation. Photos were analyzed for torsion by ImageJ software (ImageJ 1.54f, National Institute of Health, Bathesda, MD, USA). The disc-foveal angle was calculated for ocular torsion. Disc-foveal angle was defined as the angle formed between a line passing through the center of the optic disc to the fovea and another horizontal line passing through the center of the disc, using fundus photographs.

A total of nineteen children were included in the study—twelve boys, mean age 9.55 ± 4.35 years, and seven girls, mean age 9.03 ± 4.3 years. An additional three patients who were operated in another institution before presentation were excluded from the study. All patients were examined for horizontal strabismus, except one with torsional strabismic amblyopia without overt horizontal strabismus. All had digital fundus photos of both eyes at presentation, and 5 of 12 also had fundus photos following strabismus operation.

The study was approved by the local Institutional Ethics Committee and adhered to the tenets of the Declaration of Helsinki. Informed consent was waived due to the retrospective nature of the study.

## 3. Results

The children’s demographic and clinical data are summarized in Table 1. Of the 19 children included, 18 had horizontal strabismus: 9 with exotropia and 9 with esotropia. One child was orthophoric with torsional strabismus. Inferior oblique overaction was detected in 16 patients, while V pattern was documented in 10. Visual acuity was reduced (under 6/12) in four eyes of four children. Twelve children underwent strabismus surgery, five of which had postoperative fundus photographs. None were symptomatic preoperatively for ocular torsion. Although extorsion was documented in 3 of the 19 children, it was measurable on fundus photos in all patients before surgery, with a mean of 8.7 ± 8.5 degrees and 8.5 ± 9.7 degrees in the right and left eyes, respectively. The mean extorsion in both eyes was 19.7 ± 10.1 degrees and improved to a mean of 15.3 ± 7.9 degrees in the children who underwent strabismus surgery. Nine children had exotropia, nine had esotropia, and one was orthophoric with amblyopia. Inferior oblique over-action was noted in 15 children, but V pattern (>15 PD) was only noted in 11. The other 4 children had only 10 PD differences in their vertical gazes. None had A pattern strabismus.

## 4. Discussion

In this study, we documented ocular torsion in children with horizontal strabismus, eso- and exotropia, and one orthophoric child with monocular amblyopia. We found that while none had subjective complaints of torsion, on clinical fundus exam three children were diagnosed with torsion, while fundus photos demonstrated ocular torsion in all cases. We found that ocular extorsion is very common in horizontal strabismus in children, in the presence of overaction of the inferior obliques, even if a V pattern is not measured. We report here that extorsion can be easily documented using fundus photos, and that the high incidence of torsion in children with horizontal strabismus should be taken into consideration when planning strabismus surgery. Ocular torsion was first reported as part of the strabismus pattern when overaction of the obliques was measured [2]. However, the lack of clinical manifestations and subjective complaints, together with difficulties in documenting torsion in the past, led to underdiagnosis of objective torsion [1]. Fundus exam of children can be difficult. Clinical exams often need to be fast and performed in suboptimal conditions when the child is not cooperative or restless. Fundus photos taken with both eyes open enable better static imaging for the further evaluation of possible torsion. While the objective motor torsion calculation is difficult to document during child fundus exams [13,14], the fundus camera can yield high-quality photos from which the fovea-to-optic disc relationship is calculated. Children with infantile strabismus often have associated oblique muscle overaction with secondary disconjugate binocular torsion [15,16] and with fundus photos it seems the rates are even higher than described decades ago. Advanced imaging using non-mydriatic fundus cameras can be easily performed in children and enable the accurate measurement and documentation of ocular torsion. The calculation of ocular torsion from fundus photographs was reported to take approximately 6 min [17]. Fundus photos may substitute the current methods taken to measure subjective ocular torsions, such as Double Maddox Rods or Lancaster red-green testing, which depend on the child’s cooperation. The use of the old Synoptophore to assess torsion was mostly abandoned since it is a time-consuming exam.

Children with both exotropia and esotropia were included in this study, in contrast to the study described by Lee et.al. which included a large cohort of children with intermittent exotropia only, all of whom had undergone surgery [11]. We found almost all children in our study had extorsion, even if overaction of the inferior obliques was not detected. Measurements of torsion in healthy children without strabismus were published by Jahtani et al. [6]. In children aged 5–15 years, with a mean age of 10.6 ± 2.5 years, the mean disc-fovea angle was 6.49 ± 3.25° (0–13°) and 5.80° ± 3.29° (0–12°), in the right and left eye, respectively [6]. In another pediatric study, 2–12 year-old children with strabismus had torsion measured in the same frequency in both exotropia and esotropia [18]. The torsion angles in the exotropia and esotropia groups were −16.35 ± 5.20 and −12.71 ± 3.16, respectively. In this study, 14 children had <5° extorsion (8 in the right eye and 6 in the left). However, in these children, the fellow eyes had >10° torsion, ranging between 7–27° in all except two. Therefore, the calculation of bilateral torsions better indicated the total torsion of the eyes. These values ranged from 6.8 to 49.6°.

The reports of cyclofusion in adults were calculated at a total mean amplitude of 16 degrees, a fusion range of +7 degrees incyclotorsion, and up to −9 degrees of extorsion [7]. These values were higher than the range considered normal in children. It was shown that low values of torsion are to be expected upon clinical investigation in non-strabismic adult individuals [7]. Values outside of the reference range may be indicators of possible binocular abnormalities or physiological variations [7]. The normal ocular torsion angle reported in the literature is –5.6° ± 3.3° [6,7].

There are significant differences between horizontal strabismus and vertical strabismus secondary to superior oblique palsy. In this study, none of the children had superior oblique palsy, neither congenital nor acquired. In superior oblique paresis, the main sign can be extorsion. However, complaints about cyclotropia are limited to acquired paralysis [19]. The difficulties in diagnosis are secondary to possible findings of cyclotropia in the normal eye and not the paretic eye, the presence of head tilt toward the involved side, or the absence of any abnormal head posture [19]. Examination of patients with unilateral superior oblique muscle palsy with Maddox double rod test shows subjective excyclotropia of the nonparetic eye in 25% [20]. This can be a very confusing and misleading sign. This phenomenon is the result of a monocular sensorial adaptation to the cyclodeviation by means of a reordering of the spatial response of retinal elements along new horizontal and vertical meridians [20]. There is inconsistency and paradoxical measurement when the non-paretic eye or both eyes are measured for extorsion, and sometimes no extorsion is measured despite superior oblique palsy [21].

Cyclodeviations differ from other strabismus forms in several aspects. Patients remain asymptomatic in spite of ophthalmoscopically demonstrable torsion of the globe around the sagittal axis [22]. These mechanisms include cyclofusion and a monocular sensory adaptation which cause the images seen by the paretic eye to appear vertically and horizontally aligned even though they do not fall on the vertical and horizontal anatomic-geometric retinal meridians [22]. Children rarely complain about diplopia due to suppression, and therefore it is not surprising that children do not complain of subjective torsion, which is commonly asymptomatic. In contrast, in adults, acquired strabismus causing diplopia is the presenting symptom of new paralytic strabismus. For example, superior oblique palsy with a torsional effect that causes diplopia is commonly reported or diagnosed due to signs such as head tilt [21]. The visual cortex must calibrate the torsion to enable stable vertical orientation under conditions of alternating monocular fixation. Therefore, a head tilt is common, even when the patient is not aware of doing so. Despite this sensory disorder, a torsional effect of inferior oblique myectomy has not been reported. The ability to regain normal torsional fusion and sensory recalibration has been shown to be very fast [1].

In strabismic children, there is no binocular vision, and cyclofusion may be influenced. The high incidence of objective extorsion should influence surgical decisions to improve surgical outcome. Strabismus surgery for horizontal strabismus includes horizontal muscle recession or resection and may involve inferior oblique myectomy. Half of the children in our study (7/12 had undergone surgery) had inferior oblique myectomy. Although torsion was not taken into consideration during muscle surgery planning, it might have influenced the surgical outcome. We measured the ocular torsion in children who had strabismus surgery and found that the reduction in torsion was also observed without operation on the inferior obliques or considering torsion in preoperative calculations. Previous studies measured the change in ocular torsion in intermittent exotropia in children, and found a significant decrease in preoperative values of the torsion after the recession of the lateral recti [11]. If binocularity is regained following surgery, it might reduce postoperative torsion too. In this study, the degree of torsion was decreased from 19.7 ± 10.1 degrees to 15.3 ± 7.9 degrees following surgery in the children who were operated on.

**Figure 1:**
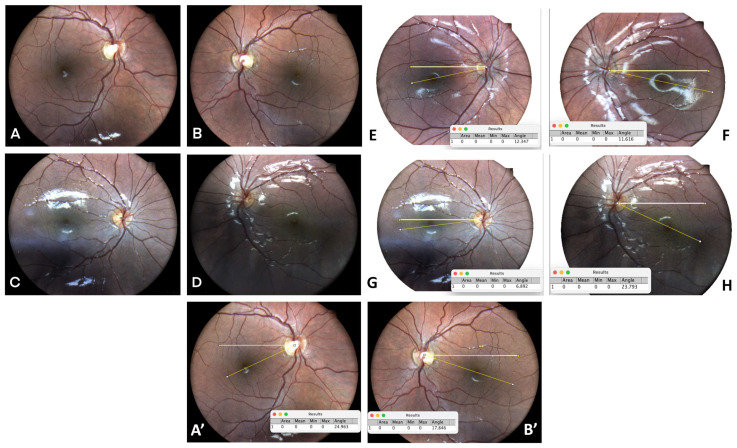
Legend to figure upper two lines: Horizontal strabismus. (**A**–**D**). Pre and post-surgery, (**A**,**C**) right eye, (**B**,**D**) left eye. (**E**,**F**). Esotropia with torsion. (**G**,**H**). Exotropia with torsion. Lower line: (**A’**,**B’**) Orthophoria with torsional amblyopia (6/24) with severe torsion (28.38 degree RE, 21.202 degrees LE, 49.582 degrees BE), calculated by Image J 1.54f. The child had no horizontal strabismus, but abnormal orbits due to craniosynostosis.

Amblyopia can be secondary to strabismus, anisometropia, or deprivation. Three children had monocular amblyopia in this study. However, another child had amblyopia without strabismus. This case represents unexplained amblyopia that was possibly associated with “microstrabismus”. We theorize that the torsional deviation, secondary to craniosynostosis and abnormal orbit size, might have led to his amblyopia. Torsion is commonly associated with strabismus and may provide an impediment to fusion in acquired cases. In this study, we suggest that extorsion might be the undiagnosed reason for his mild to moderate amblyopia (Figure 1).

In this study, we included both children with esotropia and exotropia, with or without amblyopia. The position of the eye (for torsion measurement) was not influenced by stereopsis and could be photographically documented even when suppression or amblyopia existed. This was based on the findings that in the presence of vertical strabismus (4th nerve palsy), occlusion of one eye will not change the torsional position of the eye if this is the dominant preferred eye [15]. Eye preference also may influence the torsional measurement. Surprisingly, even in a monocular photograph of the fundus, when both eyes were open the torsion could be easily detected. There was a significant correlation between the dominance of ocular torsion and fixation preference [11]. Even in paralytic strabismus, ocular torsion may occur in the paretic or non-paretic eye, depending on eye preference.

The findings that torsional strabismus is common even in the absence of a clear V pattern, and is influenced by surgery of the horizontal muscles, might contribute to the understanding of amblyogenesis and influence decisions such as when to operate and how to predict successful response to surgery. Clinical examination of ocular torsion has become part of strabismus diagnosis and management [1]. However, the clinical manifestations of subjective and objective torsion are frequently conflated or disregarded, creating diagnostic confusion and therapeutic dilemmas [1].

The development of deep learning models can aid in diagnosis and measuring the direction and amount of intorsion and extorsion [23]. Sensory torsional recalibration was recently described as a critical mechanism to overcome objective torsion [1]. This sensory torsional control is attributable to the ability of the human visual cortex to recalibrate subjective torsion and prevent vertical disorientation. There is no doubt that measuring objective torsion before surgery improves the surgical success, and when ignored the visual cortex will overcome the remaining torsion [1].

An additional aspect of artificial intelligence application in this field can be seen in fundus photo analysis. The use of fundus photographs had already been already reported in 1986, when photorefraction was found to be a reliable, simple, and cheap tool with high sensitivity and specificity in screening for strabismus [24]. With advances in artificial intelligence and deep learning models, reports of the use of fundus photos to develop and optimize diagnosis have accumulated, mainly in the field of diabetic retinopathy [25]. Being increasingly accessible [25], it should be introduced for torsional strabismus analysis as it is also based on simple retinal photo calculations.

In 1983 Kushner et al emphasized the role of fundus photography in the diagnosis of strabismus [26]. It is not surprising that the study evaluated the objective presence or absence of torsional strabismus [26]. As torsional strabismus has been a fascinating subject over decades, the use of advanced technology mainly supports the traditional Kushner findings. Even then, the presence or absence of objective ocular torsion as seen in fundus photographs had a sensitivity of 0.86 and a specificity of 0.96 for diagnosing the presence of oblique dysfunction, and a sensitivity of 0.96 and specificity of 0.83 for diagnosing the presence of normal oblique function [26]. A recent study in 2022 [18] showed that children with ocular torsion on fundus photography had a high prevalence of strabismus. Similar to the findings in our study, esotropia and exotropia were studied. There were no differences in the frequencies of exotropia and esotropia based on the torsion type [18]. Extorsion was more frequent than intorsion, with a greater extorsion angle in exotropia than in esotropia [18]. Since then, technology has significantly improved over the recent years and is still improving. Fundus photos can be taken without mydriasis in children almost as young as 4 years old who are able to sit and look into the fundus camera. As declared by Brodsky, an examination of ocular torsion is therefore integral to strabismus examination and critical for surgical decision making [1]. Although many aspects of its effects on vision remain controversial, any surgical plan should seek to improve significant degrees of torsion, or at the very least not make them worse. In the torsionally asymptomatic patient with acquired strabismus, treating torsion may be considered analogous to placing a patient with diplopia back into the center of their field of single binocular vision in the primary position to minimize future symptomatology.

Optical coherent tomography (OCT) is an additional tool to photograph the retina, and it has the ability to demonstrate the internal layers. In a recent study by Yamadera et al., the Glaucoma Module Premium Edition (GMPE) software for the SPECTRALIS OCT was used to automatically track the anatomic centers of both the fovea and the optic disc [17]. This method was used to measure ocular torsion before and after strabismus operation in adults [17]. The measurements were taken before and one day after surgery. There was a high correlation between the OCT and the non-mydriatic photos, but less correlation with the synoptophore testing [17]. This was in correlation with underdiagnosed torsion due to minor subjective complaints. The variability in objective measurements was demonstrated as the synoptophore measurement based on patients’ responses were less than those with the objective measurements of both fundus photos and OCT [17]. Both methods were found to be helpful in detecting cyclotropia.

In this study, only a few cases were measured after surgery. Those measurements were taken at the end of follow-up, at least one month after surgery. In a study by Sharma et al., measurements were taken 3 months after the strabismus operation [27]. It is much more challenging to persuade a child to perform accurate measurements one-day post-operation, as presented in the Yamadera study [17]. In both studies, strabismus surgery improved cyclotorsion. In vertical diplopia, this is the aim of the operation. However, in horizontal strabismus, as presented in our study, the incidence of torsion was surprisingly high. There was a significant reduction in torsion after surgery [17]. As expected, and even as seen in this study, we noted reduced torsion only for horizontal strabismus when the oblique muscles were operated. The interpretation of ocular torsion and its clinical consequences is a nuanced operation. At the outset, it is critical to distinguish subjective or sensory torsion (perceptual tilt of the visual world) from objective or motor torsion (the degree of torsion visible to the examiner) [13,14].

This study included only 19 children. An additional three children presented after being operated on in another hospital and were therefore excluded. It should be noted that children as young as 4 years old were also included in the study, emphasizing the importance of torsion detection in fundus photos. The majority of the horizontal strabismus presented with inferior oblique overaction. Only a minority had fundus photos after surgery (5/12). However, all showed a reduction in extorsion. The increased detection of torsion might influence strabismus surgery planning and may be associated with operation success rates [10].

We have shown that multiple ocular findings can be associated with torsion, such as inferior oblique overreaction with or without V pattern strabismus, suppression/diplopia, and amblyopia. Therefore, it is important to evaluate such patients for ocular torsion. Extorsion is more common than intorsion, and both are easily documented in fundus photos. Digital fundus photos should be used as part of strabismus evaluation showing ocular torsion at diagnosis, aiding preoperative decisions and surgical planning, and monitoring during follow-up.

## 5. Conclusions

In this study we have shown that extorsion was detected on fundus photos at a significantly higher rate than in clinical examination. Inferior oblique overaction was mainly associated with torsion. Although the study’s cohort size and design provide qualitative rather than quantitative data, the results are of clinical significance. The easily obtained fundus photos of children, particularly when considering strabismus surgery, may therefore have paramount importance in clinical practice and may benefit surgical planning and outcomes.

## Figures and Tables

**Table 1:** Children’s demographic, clinical, strabismus, and torsion data.

Pts. No.	Age	Sex	Strab. Type	Sub. Torsion	Obj. Torsion	RE Fundus Torsion	LE Fundus Torsion	BE Total Torsion	V Pattern	IOOA	VA	Amblyopia	Strab. Op	RE Torsion Post. Op	LE Torsion Post. Op	Post Op Torsion	Op. Details	Follow-Up (m)
1	5	F	XT	no	no	11.654	10.718	22.372	yes	R+5 L+4	6/9 6/9	0	0	N/A	N/A	N/A	NO	9
2	6	M	XT	no	yes	4.858	27.687	32.545	yes	R+4 L+4	6/7.5 6/7.5	0	0	N/A	N/A	N/A	NO	14
3	16	F	XT	no	yes	12.994	2.401	15.395	no	no	6/9 6/7.5	0	0	N/A	N/A	N/A	NO	16
4	7.5	M	ET	no	no	15.707	2.844	18.551	yes	R+3 L+5	6/9 6/24	strab	0	N/A	N/A	N/A	NO	26
5	7.5	M	ET	no	no	4.217	24.545	28.762	yes	R+1 L+5	6/12 6/7.5	strab	0	N/A	N/A	N/A	NO	26
6	6	M	ET	no	yes	24.383	4.099	28.482	yes	R+4 L+4	6/12 6/9	strab	0	N/A	N/A	N/A	NO	28
7	14.9	M	XT	no	no	6.191	11.294	17.485	no	R+3 L+3	6/7.5 6/7.5	0	1	N/A	N/A	N/A	NO	19
8	5	F	XT	no	no	7.022	−4.671	11.693	yes	R+5, L+5	6/7.5 6/7.5	0	1	4	−6.8	10.83	NO	18
9	17	M	XT	no	no	4.63	11.522	16.152	yes	R+4 L+5	6/6 6/6	0	1	4.6	12	16.24	YES	14
10	12	F	XT	no	no	5.557	4.485	10.042	yes	R+4 L+5	6/6 6/6	0	1	2.3	−3.4	5.705	NO	16
11	5.5	F	ET	no	no	15.618	2.939	18.557	yes	R+2 L+2	6/9 6/9	0	1	N/A	N/A	N/A	NO	12
12	5	M	ET	no	no	11.519	13.621	25.14	yes	R+3 L+3	6/7.5 6/9	0	1	N/A	N/A	N/A	NO	24
13	9.5	F	XT	no	no	−7.729	−12.918	20.647	no	R+3 L+3	6/7.5 6/7.5	0	1	N/A	N/A	N/A	NO	21
14	9.5	F	XT	no	no	−2.936	8.338	11.274	no	R+4 L+3	6/6 6/6	0	1	N/A	N/A	N/A	NO	3
15	14.5	M	Ortho	no	no	28.38	21.202	49.582	no	R+5 L+5	6/24 6/9	torsion	0	N/A	N/A	N/A		2
16	13	M	ET	no	no	4.958	7.394	12.352	no	no	6/6 6/6	0	1	5.4	5.1	N/A	YES	10
17	4	M	ET	no	no	8.143	2.118	10.261	no	R+2 L+2	6/6 6/6	0	1	6.7	2.7	N/A	NO	7
18	5.5	M	ET	no	no	4.946	12.839	17.785	no	R+2 L+2	6/9 6/9	0	1	N/A	N/A	N/A	NO	20
19	13.5	M	ET	no	no	4.719	2.073	6.792	no	no	6/7.5 6/7.5	0	1	N/A	N/A	N/A	NO	6

Abbreviations: M—male; F—female; XT-exotropia; ET—esotropia; Ortho—orthophoria; Strab.—strabismus; RE—right eye; LE—left eye; BE—both eye; R—right eye; L—left eye; IOOA—inferior oblique overaction; Post Op—post operation; Follow up (m)—months; N/A—not applicable.

## Data Availability

The data presented in this study are available on request from the corresponding author.
